# Polyvinyl Alcohol-Chitosan Scaffold for Tissue Engineering and Regenerative Medicine Application: A Review

**DOI:** 10.3390/md21050304

**Published:** 2023-05-17

**Authors:** Kavitha Ganesan Nathan, Krishnamurithy Genasan, Tunku Kamarul

**Affiliations:** 1 Department of Orthopedic Surgery, Faculty of Medicine, University Malaya, Kuala Lumpur 50603, Malaysia; kavitha.gnathan@um.edu.my; 2 Department of Physiology, Faculty of Medicine, University Malaya, Kuala Lumpur 50603, Malaysia; 3 Advanced Medical and Dental Institute (AMDI), University Sains Malaysia, Bertam, Kepala Batas 13200, Malaysia

**Keywords:** polyvinyl alcohol, chitosan, scaffold, tissue engineering, regenerative medicine

## Abstract

Tissue engineering and regenerative medicine (TERM) holds great promise for addressing the growing need for innovative therapies to treat disease conditions. To achieve this, TERM relies on various strategies and techniques. The most prominent strategy is the development of a scaffold. Polyvinyl alcohol-chitosan (PVA-CS) scaffold emerged as a promising material in this field due to its biocompatibility, versatility, and ability to support cell growth and tissue regeneration. Preclinical studies showed that the PVA-CS scaffold can be fabricated and tailored to fit the specific needs of different tissues and organs. Additionally, PVA-CS can be combined with other materials and technologies to enhance its regenerative capabilities. Furthermore, PVA-CS represents a promising therapeutic solution for developing new and innovative TERM therapies. Therefore, in this review, we summarized the potential role and functions of PVA-CS in TERM applications.

## 1. Introduction

Tissue engineering (TE) is an interdisciplinary field that uses the principles of engineering and biological sciences to create biological substitutes that can reinstate, sustain, or improve tissues or organs functionality in the body [1]. It provides a combination of scientific disciplines, including cell biology, medicine, and engineering, to design systems that can contribute to the development of novel cells and tissues [2]. On the other hand, regenerative medicine (RM) is a field that integrates knowledge from multidisciplinary basic research to develop clinical interventions that can substitute or rejuvenate human cells and regrow damaged tissue to restore its normal function [3]. It uses various approaches, including cell-based treatment, gene therapy, immunomodulation, nanomedicine, and TE itself, to stimulate the repair or regeneration of organ(s) [4].

Tissue engineering and regenerative medicine (TERM) were explored for over 30 years. Due to their similar goals, these two fields converged in recent years and became a potential strategy to satisfy the future requirements of patients [5]. Tissues in the human body have a limited capacity to repair or regenerate. This presents a challenge that is often difficult for clinicians to overcome. Therefore, TERM relies on various strategies and techniques to achieve its goals. The most prominent strategies are the use of scaffolds [6,7,8], cells [9,10,11], and growth factors [12,13,14]. This review focuses on the scaffold polyvinyl alcohol (PVA)-chitosan (CS) and its multiple functions in TERM applications. We also discuss the challenges and limitations associated with its use.

### 1.1. An Overview of PVA and Chitosan

Hydrogels are three-dimensional (3D) systems composed of hydrophilic polymeric materials, which exhibit a structure conducive to water absorption. This feature enables one to retain significant amounts of water. Their high-water content, softness, and porosity make them resemble living tissue and exhibit excellent biocompatibility [15,16]. They are also sensitive and intelligent enough to respond to environmental stimuli such as ionic strength, temperature, pH, electric fields, enzymes, etc. [15]. Hydrogels can be prepared from natural or synthetic polymers or their blends to produce enhanced characteristics of materials than starting polymers [17].

Polyvinyl alcohol (PVA) is a synthetic, biocompatible polymer extensively studied for developing scaffolds owing to its high strength, resistance to deformation under load, capacity for water retention, and porous architecture [18]. At the same time, chitosan (CS) is a biocompatible polysaccharide that is semi-crystalline and has a linear structure. CS is derived from the exoskeletons of crustaceans [19]. The most intriguing property of CS is its ability to be processed into porous structure that is well-suited for cell transplantation and tissue regeneration applications [20]. Although CS-based hydrogels are promising for biomedical applications, their mechanical characteristic was often a drawback [21]. Therefore, a combined material such as nanoparticles, biopolymers, and synthetic polymers could solve this challenge by offsetting the limitations of one another and creating an ideal biomaterial.

**Figure 1:**
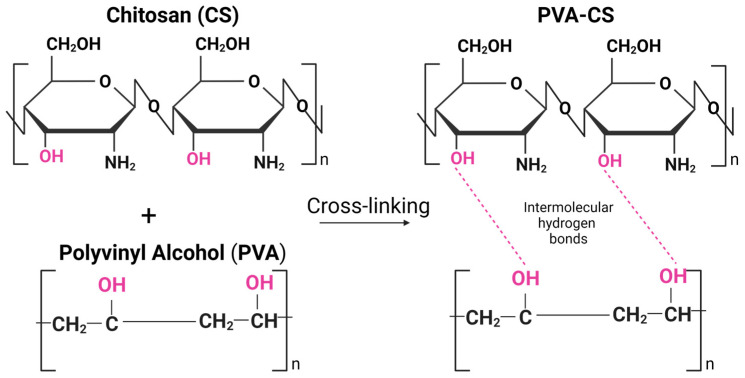
Intermolecular hydrogen bonds between chitosan (CS) and polyvinyl alcohol (PVA) polymeric chains. Created by BioRender.com (accessed on 23 April 2023).

Incorporating PVA into the CS-based hydrogel matrix can enhance its mechanical properties. Several recent studies focused on the combination of PVA and CS, and the combination was shown to have good mechanical and chemical properties due to their unique intermolecular interactions based on their chemical structure and physical properties [22,23,24]. PVA is a water-soluble synthetic polymer with a high degree of hydrogen bonding between its hydroxyl groups (-OH), while CS has amino groups (-NH_2_) in its chemical structure, which can form hydrogen bonds with the hydroxyl groups of PVA (Figure 1). Due to hydrogen bonding and electrostatic interactions, the PVA-CS mixture can also form physical crosslinks that can contribute to the unique properties of the material, such as increased tensile strength, toughness, and stability in aqueous environments [25].

In addition, PVA-CS blends exhibit high blood compatibility [26] and have the potential to serve as effective drug delivery platforms [27]. Therefore, PVA–CS combinations are extensively investigated in membrane filtration, dye adsorption, packaging, and biomedical applications [28,29].

### 1.2. Physical and Biological Properties of PVA-CS Hydrogel

A biomaterial for TE should be biocompatible because the interplay between the implanted growing cells and the biomaterial significantly influences the development of new tissue. Biomedical material should not cause harm, including toxic, injurious, or immunological responses in living human tissue, blood, or the immune system [30]. PVA blends hydrogels were used in various combinations and compositions with either chemical or thermal cross-linking agents such as glutaraldehyde [31,32], sulfosuccinic acid [33,34], succinic acid [33], glyoxal [35], dianhydrides [36], maleic acid [37], sodium hexametaphosphate [38], citric acid [39], trimetaphosphate [38], and formaldehyde [26]. The existence of residual cross-linking agents may result in detrimental adverse reactions. Moreover, the drug-cross-linker interactions can give rise to the creation of noxious or ineffectual derivatives [40]. Therefore, biocompatibility and toxicity tests are necessary before biomaterials can be used for clinical applications.

Freeze/thaw processing is another method to produce hydrogel without using chemical cross-linkers [41]. PVA hydrogels prepared by freeze/dry cycles are excellent biomaterials owing to their water swelling capacity, rubbery elasticity, non-toxic, non-carcinogenic and are well tolerated by the body [41]. Several studies focused on combining PVA with CS to produce soft, swellable, and flexible PVA-CS hydrogels using this method [23,42,43].

PVA-CS hydrogel has unique physical and biological properties, making it a popular choice for biomedical applications. This combination of soft, elastic, and flexible nature makes it suitable for TE and wound-healing applications [44,45,46]. In addition, its high-water absorption capacity makes it an excellent choice for wound dressings [44] and drug delivery applications [47]. The water content of the hydrogel can be adjusted by varying the ratio of PVA and CS, which affects the mechanical strength and swelling properties [48]. In addition, CS has antibacterial properties that can help preventing infections during wound healing. Therefore, due to its unique physical and biological properties, PVA-CS was introduced as a promising material for various biomedical applications.

### 1.3. Characterization Techniques for PVA-CS

PVA-CS hydrogels with different ratios underwent evaluations for their physiochemical and mechanical traits, cytotoxicity, and biocompatibility [24,49]. Various approaches can be used to study the properties of PVA-CS hydrogels. The most common techniques include; (i) the swelling behavior can be studied by measuring its weight before and after swelling, (ii) the water content can be determined by weighing before and after drying, (iii) the mechanical properties such as elasticity, tensile strength, and modulus can be determined by various methods including shear, tensile, and compression tests, (iv) the transparency and optical properties can be analyzed using UV spectroscopy and transmission electron microscopy (TEM), (v) the morphology can be analyzed using TEM or scanning electron microscopy (SEM), (vi) the biocompatibility can be tested by performing cell culture assays for cell viability, proliferation, and differentiation, (vii) the antibacterial properties can be evaluated using disc diffusion assays, colony forming units (CFU), or minimum inhibitory concentrations (MIC) assays, and (viii) the biodegradability can be evaluated by measuring the weight loss or degradation of the hydrogel over time [24,50,51,52].

## 2. Applications of PVA-CS Hydrogel

The biocompatible and biodegradable properties of PVA and CS make them attractive biomaterials for the application in the field of TERM [44]. Many studies showed that the combination of PVA and CS has several desirable properties that make them suitable for various medical applications [53,54]. Therefore, this review is a brief overview of the recent application of the PVA-CS scaffold in TERM Table 1.

### 2.1. Tissue Engineering

TE is a vital area of biomaterials application as the various approaches can be used to treat abnormalities in tissues and organs [65,66]. TE aims to create a 3D cell-containing scaffold implanted into the body to treat a disease or repair damage [67]. The standard in vitro culture system cannot mimic the intricacies of the cellular microenvironment and seldom facilitates the integration of cells into fully functional tissue. Therefore, providing an appropriate scaffold of a diverse range of natural and synthetic materials will lead to the development of functional tissue.

In recent years, PVA-CS was widely used as a TE scaffold. PVA-CS scaffold could be a good platform for tissue regeneration alone or in combination with other polymers and cellular components [53]. The scaffold provides a 3D architecture that imitates the original tissue’s extracellular matrix (ECM), providing a favorable environment for stem cell growth and differentiation [68]. Stem cells are undifferentiated cells that can develop into distinct types of specialized cells. Depending on the application and regenerated tissue, different types of stem cells can be integrated with biomaterial scaffolds. The combination of stem cells and biomaterial scaffolds presents a promising approach for both in vitro and in vivo TE applications [68].

PVA-CS scaffold is a promising biomaterial for cartilage repair, while mesenchymal stem cells (MSCs) are emerging as a better option due to their unique properties and potential to promote tissue regeneration and repair [69]. MSCs are a type of adult stem cell that can differentiate into multiple cell types, including chondrocytes, adipocytes, osteocytes, etc. When MSCs are introduced into damaged cartilage tissue, they can help stimulating the repair process and promote the growth of new cartilage cells. One of the advantages of using MSCs for cartilage repair is that they can be obtained from a variety of tissues, including bone marrow (BM), adipose tissue (AT), and umbilical cord tissue (UCT). This means that MSCs can be easily isolated and propagated in vitro, making them a readily available option for cartilage repair and cell-based therapy [70]. In addition, MSCs were shown to secrete various growth factors and cytokines that can promote tissue regeneration and repair. These factors can help stimulate new blood vessel growth, reduce inflammation, and promote the growth of new cartilage cells [69]. This could be the reason why MSCs are often combined with natural or synthetic hydrogels to enhance biocompatibility, biodegradability, and cellular response.

**Figure 2:**
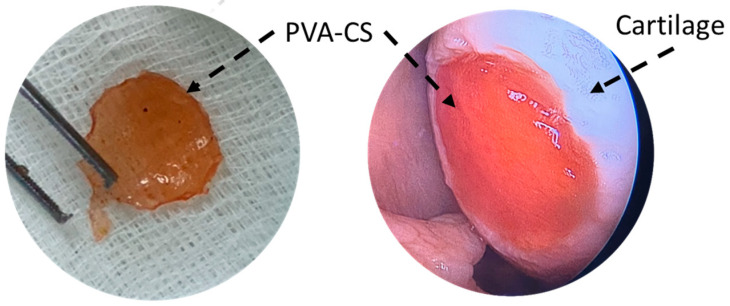
PVA-CS was implanted at the cadaveric knee cartilage defect using a minimally invasive arthroscopic technique.

In 2015, Dashtdar et al. investigated whether MSCs seeded in PVA-CS hydrogel could result in comparable or even better cartilage healing than that of previously established alginate-transplanted model. This study confirmed that the PVA-CS-MSCs construct leads to comparable treatment outcomes in the rabbit cartilage defect model, thus suggested for for clinical applications in cartilage regeneration [49]. In extension to this study, our group implanted the PVA-CS in a cadaveric knee cartilage defect using a minimally invasive arthroscopic technique as part of the technical validity prior to our clinical trial study (Figure 2). In 2019, Peng et al. demonstrated that the hydrogel PVA-CS provided an excellent surface for rabbit bone marrow mesenchymal stem cells (rBM-MSCs) adhesion and proliferation. In addition, this group demonstrated that PVA-CS caused no cytotoxicity and achieved the best cartilage repair compared to scaffold alone in an in vivo rabbit model [24].

Nour-Eldeen et al. established a scaffold that allowed adipose-derived mesenchymal stem cells (ADSCs) to proliferate and differentiate into chondrocyte-like cells using PVA-CS nanofiber scaffolds [55]. Characterization of seeded cells, including cell morphology, analysis of surface markers, and chondrogenic differentiation, were studied in vitro. This study suggests that using PVA-CS nanofiber scaffolds had a promoting effect on chondrogenic differentiation of ADSCs, as demonstrated by significant upregulation of aggrecan and collagen type II alpha 1 Chain (COL2A1), suggesting PVA-CS-ADSCs nanofiber scaffolds can potentially be used to improve the pathophysiology of osteoarthritis (OA). Various types of PVA-CS nanofibers were investigated for biomedical applications. PVA-CS nanofibers can be synthesized through different electrospinning techniques, resulting in different fiber morphologies and properties [71]. Many studies incorporated various biological and polymeric materials into PVA-CS nanofibers to improve their properties. 

Moreover, the osteoconductive and tissue regeneration performance of the fabricated scaffold was demonstrated with and without AT-MSCs in vivo rat model [56]. Abazari et al. incorporated hydroxyapatite (HA) and platelet-rich plasma (PRP) into PVA-CS to study MSCs survival and osteogenic differentiation potential in vitro. The in vivo study showed that PVA-chitosan-HA(PRP) successfully repaired bone defects to a considerable extent. However, when MSCs were seeded onto PVA-chitosan-HA(PRP), the defects were almost filled. Therefore, it can be inferred that PVA-chitosan-HA(PRP) alone or with cultured stem cells has a promising option as an efficient bone implant.

Recently, Wee et al. investigated the impact of transforming growth factor-beta 1 (TGF-β1) and -β3 on the chondrogenic differentiation of rBM-MSCs, grown on the PVA-CS-PEG (polyethylene glycol) scaffold in comparison to pellet cultures [72]. The study reported that utilization of the PVA-CS-PEG scaffold improved both the proliferation and chondrogenic differentiation of rBM-MSCs. However, no significant differences were observed between the cultures supplemented with or without TGF-β, suggesting no effect of TGF-β1 and TGF-β3 in chondrogenic differentiation. Enhanced cell proliferation observed in PVA-CS-PEG scaffolds may be attributed to the positive charge of chitosan, which facilitates the adhesion and proliferation of BM-MSCs on the scaffold [73]. Moreover, the PVA-CS-PEG scaffold offers a beneficial 3D porous structure that enables high-density cell proliferation of BM-MSCs within the scaffold due to its large surface area-to-volume ratio [72].

Mohammadi et al. developed a novel 3D nanofiber hybrid scaffold of poly(ε-caprolactone), PVA, and CS for bone tissue engineering using MSCs via a multi-jet electrospinning method [57]. The scaffolds’ chemical, physical, and structural properties were investigated to determine their impact on the differentiation of MSCs into osteoblasts and the proliferation of the differentiated cells. SEM microscopic images of MSCs seeded and differentiated on the scaffold showed that the cells attached, permeated, and uniformly distributed within the construct. Additionally, the expression of osteoblastic differentiation markers, including osteocalcin (OCN), osteopontin (OPN), alkaline phosphatase (ALP), and bone sialoprotein (BSP) exhibited an upregulation in constructs cultured in osteogenic media suggested that nanofibrous scaffolds may be favorable for TE [57]. The mechanisms by which PVA-CS nanofibers scaffold promotes osteoblasts differentiation and proliferation of MSCs are multifactorial and involve both physical and chemical cues. The 3D structure of the nanofibers scaffolds that mimic the natural ECM of bone tissue allows for the adhesion and proliferation of MSCs, providing a suitable microenvironment for osteoblast differentiation. Additionally, the high surface area-to-volume ratio allows for the efficient exchange of nutrients and waste products between the cells and the culture medium [58,59].

Interestingly, CS was shown to stimulate osteoblast differentiation and mineralization. Mathews et al. demonstrated the osteogenic potential of CS in a 2D culture system. This study presented novel findings indicating that CS enhanced mineralization by upregulating the genes involved in mineralization as well as calcium-binding proteins such as OPN, Integrin binding sialoprotein (IBSP), Collagen type I alpha 1 chain (COL1A1), ALP, and OCN [74]. Chen et al. developed chitosan nanofibers to investigate their impact on osteoblast maturation and the underlying mechanisms of action in vitro [75]. This study reported that chitosan nanofibers could promote the growth and development of osteoblasts by regulating the expression of genes associated with osteoblasts function, including OPN, OCN, and ALP through the Runt-related transcription factor 2 (RUNX2) pathway [75].

For cardiovascular TE, PVA/CS or PVA-CS was used as a coating material for cardiovascular stents to improve their biocompatibility and reduce the risk of restenosis [76]. In addition, PVA/CS was studied as a potential material for artificial blood vessels and heart valves TE [77]. Research showed that the combination of PVA and CS can yield a composite material with enhanced properties and biocompatibility compared to any single polymer. For example, a study published in the Journal of Biomaterials Science, Polymer Edition, found that a PVA-CS composite coating on a cardiovascular stent reduced the risk of restenosis and improved endothelial cell proliferation compared with a stent coated with PVA or CS alone. Karami et al. reported that PVA-CS composite coating on a cardiovascular stent reduced the risk of restenosis and improved endothelial cell proliferation compared to a stent coated with only PVA or CS [78].

### 2.2. Drug Delivery System

Hydrogel delivery systems are used clinically and can provide therapeutically beneficial effects. The use of hydrogels allows for the precise control of the time and location of therapeutic agent delivery, including small-molecule drugs, macromolecular drugs, and cells [47].

PVA-CS was used as a drug delivery system for various therapeutic agents in TE. This combination was shown to enhance drug solubility and stability, increase drug uptake by cells, and improve drug release kinetics. The hydrophilic nature of PVA and the cationic characteristic of CS provides an ideal environment for drug loading and delivery.

In drug delivery, PVA-CS can be used in multiple forms, such as nanoparticles, microparticles, and hydrogels. The different forms have different advantages and can be tailored to meet specific drug delivery requirements. Mahato et al. developed PVA-CS lactate hydrogel and investigated it as a matrix for the continuous and gradual release of hydrophilic drugs [54]. The developed PVA-CS lactate was cross-linked, and freeze-bound water was measured to analyze the cold crystallization properties. Cell adhesion, cytotoxicity, hemolysis, and drug release properties were also investigated. In vitro cell viability of L929 cells showed that PVA-CS lactate hydrogels were compatible with cells and improved cell adhesion. Moreover, the release of ciprofloxacin from the drug-loaded PVA-CS lactate hydrogels inhibited the growth of E. coli, which provided antibacterial activity under physiological conditions [54]. Fathollahipour et al. synthesized a series of hydrogel by blending PVA-CS and adding different amounts of graphene oxide (GO) to develop composite hydrogels [60]. In this study, the drug release profile and kinetics of the drug were studied to predict the mechanism of drug release.

In recent years, PVA-CS nanoparticles were used to encapsulate various drugs, including anticancer drugs, antibiotics, and anti-inflammatory agents. They were shown to increase drug bioavailability and potentially target specific cells or regions. In 2011, Parida et al. included Cloisite 30B in the formulation of PVA-CS as a matrix material component, and curcumin was prepared at various concentrations and loaded with PVA-CS/C 30B nanocomposites to investigate the in vitro drug delivery system for anticancer drugs [61]. They studied the kinetics of the drug release in order to ascertain the type of release mechanism. The kinetics results showed that the drug release was much more significant in the basic medium than in the acidic medium [61].

Shagholani et al. has improved the interaction between PVA and CS hydrogel by magnetite nanoparticles, making them a favorable option for drug delivery and clinical applications [79]. They synthesized magnetite nanoparticles by co-precipitation with ultrasound and then coated them with CS. The CS-coated magnetite nanoparticles were then coated with PVA. These modified nanoparticles present minimal protein adsorption, making them feasible for evading opsonization during clinical applications and drug administration [79]. Cui et al. fabricated PVA-CS nanofibers containing ampicillin sodium using the electrospinning technique. This study reported that the drug release studies, and kinetic analysis of the drug delivery system fitted to the Korsmeyer–Peppas model [62].

Microparticles, microcapsules, and microspheres are common constituents of multiparticulate drug delivery systems. Microparticles are spherical particles ranging in size from 1 to 1000 µm and are used as multiparticulate drug delivery systems to improve efficacy, tolerability, and patient compliance [80]. Microparticles from PVA-CS were used to sustain drug release over an extended period. These particles were loaded with drugs and implanted into the patient’s body for controlled drug delivery [81]. Morelli et al. fabricated PVA-CS microparticles by emulsification for the purpose of encapsulation and controlled release under pH conditions. This study developed a novel technique combining cross-linking with emulsion formation to produce particles with different release profiles based on polymer composition and cross-linking. The study reported that when negatively charged drugs like sodium salicylate are encapsulated, the release of the drug is delayed, and it impacts the selective release under acidic pH conditions [82].

In 2010, the hydrogel PVA-CS was developed to deliver insulin through the nasal cavity [83]. The PVA-CS hydrogels were prepared with different formulations, and the pH was adjusted to a near-neutral value of 1.0 M NaHCO_3_. Insulin was incorporated into the formulated delivery system, resulting in a final solution with a concentration of 1 IU of insulin per 200 µL. The in vitro insulin release assay showed that glucose levels were maintained for 6 h, while in the in vivo experiment, the greatest reduction was observed 4 h after administration [83]. This suggests that slow release was achieved via the PVA-CS network. In 2017, a similar study was conducted to evaluate the potential of PVA-CS microspheres as a vehicle for insulin drug delivery via intranasal administration [84]. The authors developed different formulations, and morphological analysis of the optimized formulas showed that the size range was between 200 nm to 2 µm. The in vitro study showed that microspheres from PVA-CS exhibited immediate, sharp, and erratic drug release, while the in vivo investigation in rats demonstrated a reduced drug release rate and better mucoadhesive properties [84]. Thus, using PVA-CS in drug delivery systems produced favorable outcomes.

### 2.3. Wound Healing

PVA-CS was extensively studied for its potential use in wound healing, as its water retention capacity and antibacterial activity indicate that it is a perfect material for wound treatment [44,45]. CS was shown to have antimicrobial properties due to its ability to interact with bacterial cell membranes and disrupt their structure [84]. The antimicrobial activity of CS may be due to several factors, including its positive charge, which allows it to bind to negatively charged bacterial cell membranes, and its ability to form a gel-like that can physically block the growth and spread of bacteria [84,85,86]. For this reason, it was used in various forms, including hydrogel patches, films, nanofibers, and scaffolds.

PVA-CS hydrogels and films are most used as wound dressings because they can retain water, which is critical for healing. This hydrogel absorbs exudate and creates a protective barrier that shields the wound from external contaminants. Several studies reported the antibacterial activity and healing properties of PVA-CS loaded with other active ingredients [45,46,63,87,88]. Niranjan R. et al. combined PVA-CS with curcumin (CUR) and obtained as PVA-CS-CUR patches by gel casting showed antibacterial activity against the most prevalent strains found (*Escherichia coli*, *Pseudomonas aeruginosa, Staphylococcus aureus*, *Bacillus subtilis*) in wound sites [46]. Furthermore, in vivo studies in albino Wistar rats on wound healing ability showed that this patch has an excellent wound healing ability and can treat all kinds of epidermal damage. Similarly, Gutha et al. developed PVA-CS-zinc oxide (ZnO) beads as a novel wound-healing agent that exhibits antibacterial properties. The antibacterial activity against *Escherichia coli* and *Staphylococcus aureus* was evaluated by the inhibition method, and the wound healing properties were tested in mice skin. The PVA-CS -ZnO showed excellent antibacterial and wound-healing activity, suggesting its potential use for wound-healing applications [45].

High cell proliferation capacity is critical for wound healing. Lin et al. reported that the combination of PVA, CS, and dextran exhibited high cell proliferation ability, making them ideal for wound dressing [63]. Recently, Feng et al. reviewed that CS plays a vital role in wound healing [89]. Wound healing processes generally involve four crucial phases: hemostasis, inflammation, proliferation, and skin remodeling. The initial three stages rely significantly on the involvement of CS during the hemostasis stage. CS helps prevent bleeding by promoting platelet and red cell aggregation and preventing fibrin disintegration. In the inflammation stage, CS helps eliminate microorganisms from the wound and finally increases skin proliferation by promoting the growth of granulation tissue in the proliferation stage [89].

Due to their high surface area and ability to mimic natural tissue structure, PVA-CS nanofibers can be used as wound dressings. Nanofibers can increase cell adhesion and migration, critical for wound healing. PVA-CS nanofibers can also be loaded with active ingredients to improve wound healing. Electrospun nanofibers are well suited as wound dressing materials because they have a high surface area ratio, variable pore size distribution, and oxygen permeability [64]. Moreover, the morphology of electrospun nanofibers is comparable to skin ECM, which stimulates cell adhesion, migration, and proliferation [64,90,91] Campa-Siqueiros et al. prepared electrospun gelatin (G) and PVA-CS nanofibers and studied their physicochemical properties and antimicrobial activity [91]. They reported that PVA-CS-G could be used as a wound dressing and combined with common bioactive chemicals or growth factors for its sustained release in treating chronic diabetic patients [91]. In contrast, Liu et al. used the solution-blowing method to prepare hydrogel nanofiber mats from PVA-CS with various ethylene glycol diglycidyl ether (EDGE) content as cross-linker [92]. SEM, FTIR, and X-ray photoelectron spectroscopy (XPS) results suggested that the PVA-CS hydrogel nanofiber mats had both the advantages of a hydrogel and a fiber mat, including excess exudate absorption, facilitation of a moist wound healing environment, permitting gas exchange, and displaying strong antibacterial properties [92].

Fatahian et al. developed a hybrid fiber mat through a co-electrospun hybrid of PVA, CS, and silk fiber mats. The hybrid fiber mat characteristics, including porosity, degradability, pore size, tensile strength, and hydrophilic properties for wound healing, were investigated in vitro and in vivo by localizing BMMSC keratinocytes on the mat [87]. Compared to PVA alone and the fiber PVA-CS, incorporating mixed CS and co-electrospun silk into the PVA-based fiber mat showed excellent cell attachment and growth. In vivo tests also showed that the composite PVA-CS + silk fiber mat incorporating keratinocytes MSCs may promote wound healing and facilitate skin tissue generation [87].

## 3. Other Biomedical Applications

PVA-CS composite is also commonly used in other applications, including periodontal treatment [93], ophthalmic, orthopedic, cancer therapy, immunotherapy, gene therapy, and cosmetics Table 2.

### 3.1. Periodontal

Several studies showed that PVA-CS has properties that can be useful in periodontal treatment. Its biocompatibility, drug delivery, antibacterial, and wound healing properties showed promising treatment options for periodontal diseases. Dong et al. synthesized CS-decorated metronidazole (MTZ) microcapsules (CS@MTZ) and used them as cross-linkers for injectable PVA hydrogel preparation for periodontal drug delivery [100]. The study showed that PVA-CS-MTZ hydrogel is a suitable formulation for periodontal therapy due to its injectability, antibacterial efficacy, and underwater adhesion [100].

In periodontitis treatment, local administration of drugs or antimicrobial agents is an appropriate strategy when an infection is intensely localized in the pockets and does not respond well to mechanical debridement and systemic antibiotic treatment. Constantin et al. synthesized a PVA-CS film containing silver nanoparticles and ibuprofen to treat periodontal disease [94]. The film was evaluated for its biological activity, morphology, loading amount, mechanical properties, and ibuprofen release. The authors reported that the films had suitable antimicrobial properties against oral cavity pathogens and were biocompatible, as demonstrated by an in vitro study on human dermal fibroblasts, adult (HDFa) skin cell lines [94].

In recent years, TE scaffolds emerged as a potential treatment strategy for the repair and regeneration of tissue defects in periodontal disease [101,102]. A recent study published in the Journal of Dental Sciences found that PVA-CS could be an excellent flexible film for membranes used in periodontal regeneration, which can prevent fibroblasts from entering the wound and be used in periodontal regeneration surgery [93]. Dang et al. demonstrated the anti-inflammatory and osteogenic activity of PVA-CS-graphene oxide-astaxanthin nanofiber membranes in vitro. The electrospun membranes were found to stimulate a significant upregulation of osteogenic genes, osteocalcin (OCN), and RUNX2 in BMMSCs, and high expression of GPNMB in RAW26407 cells, leading to M2 polarization. These findings suggest that the nanofiber membranes can potentially enhance inflammatory dissipation and osteoblast differentiation [95].

### 3.2. Ophthalmic

PVA-CS blend was evaluated for its potential for ophthalmic applications as an ocular drug delivery system. It is used to improve drug bioavailability and residence time in the eye [96,103,104]. Under physiological conditions, the mixture can form a gel-like substance that can prolong drug release and minimize the frequency of administration. In addition, PVA-CS was investigated for its potential use in the treatment of ocular surface disorders such as dry eye syndrome. It was shown to improve tear film integrity and minimize ocular surface damage [97,105]. Therefore, PVA-CS seems to be a good system for drug delivery in ophthalmology and for treating ocular surface diseases. Further research and clinical trials are needed to evaluate its safety and efficacy in humans.

### 3.3. Gene Therapy

Gene therapy is a procedure that involves delivering genetic material to cells to treat or prevent diseases. Viral and non-viral vectors are commonly utilized to transport therapeutic genes into specific cells for effective treatment [106]. Non-viral vectors in gene delivery systems always refer to methods that do not involve viruses for gene transfer. In recent years, several studies reported the use of lipid-based nanoparticles [107], polymeric nanoparticles [108], and naked DNA [109] as a non-viral vector for gene transfer.

CS-based polymeric nanoparticles can be used as a non-viral vector for gene delivery. It has numerous advantages over viral vectors, such as safety and low immunogenicity [110]. In cancer research, several studies showed that siRNA-loaded CS-based nanoparticles are a promising therapeutic strategic [98,111,112,113]. Furthermore, CS was used as a carrier for in vivo delivery of interference agents such as siRNA [114]. Mulholland et al. used PVA-CS nanoparticles as a carrier to deliver a siRNA targeting FKBPL in both in vivo and in vitro models for angiogenesis in wound healing [115]. The in vitro results showed enhanced cell migration and increased endothelial tubule formation, while the in vivo study in mice demonstrated increased angiogenesis and blood vessel density [115].

### 3.4. Cosmetics

PVA-CS was widely used in the cosmetics industry. A combination of PVA-CS can offer several benefits to cosmetic products, especially skin and hair care products [116,117]. Since PVA-CS is a water-soluble polymer blend, it can improve the hydration properties of cosmetic formulations. Thin film formation on the skin can help lock in moisture and prevent dryness [116,117].

Castor et al. synthesized four distinct composite films composed of PVA-CS and tea tree (*Melaleuca alternifolia*) essential oil and tested them on Wistar rats through subdermal implantations [99]. This study indicated that the composite films exhibit excellent thermal and mechanical stability and simulated body fluid stability. These were confirmed by mechanical and thermal analyses when there was a rise in Young’s modulus and decomposition temperatures. Furthermore, the biocompatibility of the films was found to be like that of porcine collagen and with higher tea tree essential oil showing more significant signs of resorption [99].

## 4. Challenges and Limitations

**Figure 3:**
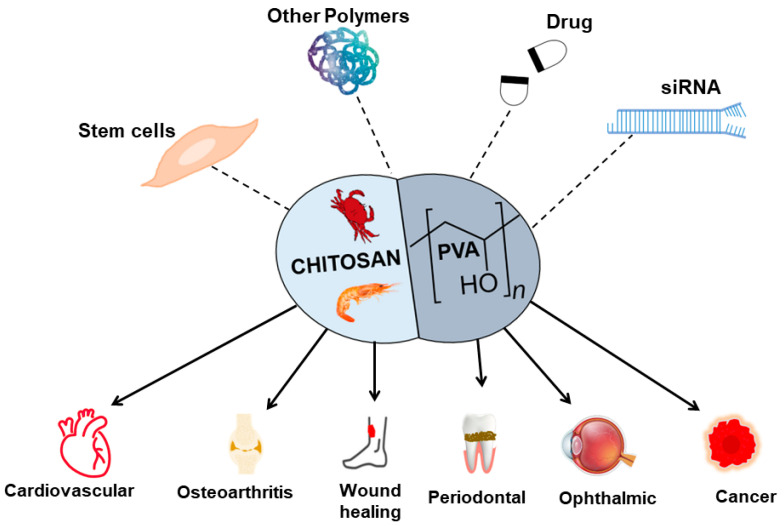
An overview of the PVA-CS combined with other materials and their application in TERM.

PVA-CS showed potential material for TERM (Figure 3), but there are also some challenges and limitations associated with its use. Although the mechanical properties of PVA-CS can be modified, they may not be sufficient for all TERM applications, as the material can also degrade over time, which can affect its mechanical properties and lead to loss of structural integrity [118]. Although PVA-CS was shown to be biocompatible [18], further studies are needed to evaluate its long-term safety and efficacy in vivo.

Fabrication of the PVA-CS scaffold itself may be challenging due to difficulties in optimizing the pore size and porosity of the scaffold. Poor pore size and porosity of a scaffold can impair cell adhesion, proliferation, and differentiation. The shape of the scaffold can influence its porosity and pore size in several ways. For example, a flat, planar scaffold may have lower porosity than a 3D scaffold with more complex geometry, such as a cylindrical or spherical shape. This is because a planar scaffold has a lower surface area-to-volume ratio, which limits the number of pores created in the material.

On the other hand, a more complex-shaped scaffold may provide a greater surface area-to-volume ratio, allowing a larger number of pores to form [119,120]. In addition, these scaffolds must be sterilized before being used in medical and clinical applications. Some sterilization methods may affect the mechanical and chemical properties of the scaffold [121].

PVA-CS nanoparticles are pH-dependent [98], which means that their physicochemical properties change with the change in pH. Therefore, their stability and functionality can be affected by changes in the pH of the surrounding environment.

The limited availability of CS, which is derived from crustacean shells, can increase the cost of production. Additionally, PVA-CS must undergo rigorous testing and regulatory approval when combined with other biologically active components and polymer materials before it can be widely used in TERM applications.

PVA-CS showed promising results in the laboratory, but producing large quantities of PVA-CS products for commercial use can be challenging. The process of synthesizing PVA-CS products can be complex and time-consuming, and scaling up the production process while maintaining desired quality and consistency can be difficult.

## 5. Conclusions

The PVA-CS blend is a promising material for TERM applications due to its biocompatibility, antimicrobial properties, and ability to support cell growth and tissue regeneration. The best is that it can be fabricated and customized to fit the specific requirements of different cells, tissues, and organs, making it a versatile tool in RM. PVA-CS nanoparticles were investigated for their potential use as drug delivery vehicles, as they can protect the encapsulated drug from degradation and improve its bioavailability. Films prepared from PVA and CS were shown to have good mechanical strength, biocompatibility, and controlled drug release. On the other hand, hydrogels based on PVA-CS can be used for TE and drug delivery due to their large water absorption capacity and 3D network for cell growth. PVA-CS can also be used in the form of scaffolds for TE and wound healing applications as they provide mechanical support and promote cell growth.

## Figures and Tables

**Table 1:** Summary of the PVA-CS applications in TERM.

Composites	Methods	Applications	Advantages	Ref
PVA-CS-BMSCs hydrogel	Freeze–thaw cycles	Osteochondral tissue repair in a rabbit model	Exhibit stable physical and chemical properties. No cytotoxicity. Promote cell proliferation.	[24]
CS/PVA/ZnO beads	Hydrothermal	Antibacterial agent and wound healing	Exhibit high antibacterial activities against *Escherichia coli* and *Staphylococcus aureus* bacteria. Fast wound healing in mice skin.	[45]
PVA-CS-Curcumin patch	Glutaraldehyde reagent crosslinker	Antibacterial agent and wound healing	Increase cell proliferation. Antibacterial activities against *Escherichia coli*, *Pseudomonas aeruginosa*, *Staphylococcus aureus*, and *Bacillus subtilis.* Wound healing ability.	[46]
PVA-CS-BMSCs scaffold	Freeze–thaw	Focal cartilage repair in a rabbit model	Cartilage repair.	[49]
PVA-CS Lactate hydrogel	Glutaraldehyde reagent crosslinker	Drug delivery	Facilitate cell adhesion. Facilitate antimicrobial activity.	[54]
PVA-CS-ADSCs nanofibrous scaffold	Electrospun	Cartilage TE	Exhibit uniform cell distribution and high biocompatibility. ADSCs were able to proliferate and differentiate into chondrogenic cells.	[55]
PVA-CS-HA(PRP) scaffold with or without MSCs	Electrospun	Osteogenic differentiation and bone reconstruction	Have a positive effect on MSCs. MSCs seeded scaffold has a huge osteoconductivity.	[56]
PVA-CS-poly(ε-caprolactone) scaffold	Multi-jet electrospun	Bone TE	Support cell attachment and osteogenic differentiation of rat MSCs.	[57]
PVA-CS- carbonated HA scaffold	Electrospun	Bone TE	Facilitate osteoblast cells to attach and proliferate. Increase cell viability.	[58]
PVA-CS-gelatinnanoHA	Paraformaldehyde reagent crosslinker	Bone TE	Promote cell proliferation and adhesion. Scaffolds containing 12.5% of nanHA were demonstrated to have high osteogenic differentiation ability.	[59]
PVA-CS- Lidocaine hydrochloride	Electrospun and glutaraldehyde reagent crosslinker	Drug delivery	Form a dual drug release delivery system. Exhibit excellent antibacterial activity against *Staphylococcus aureus and Pseudomonas aeruginosa* strains.	[60]
PVA-CS-Cloisite 30B	Glutaraldehyde reagent crosslinker	Drug delivery	Drug release was reported based on time, drug loading percentage, and pH of the medium. Drug release is more pronounced in the basic medium compared to the acidic medium. Kinetics of the drug release reported based on the non-Fickian type of mechanism.	[61]
PVA-CS nanofiber	Electrospun and glutaraldehyde reagent crosslinker	Transdermal drug delivery	Crosslinked PVA/CS composite had a lower drug release rate and smaller drug burst release. Release of ampicillin sodium fit to the Fickian diffusion mechanism.	[62]
PVA-CS-Dextran	Glutaraldehyde reagent crosslinker	Wound dressing	Present high cell proliferation. Promote thermostability and mechanical properties. Promote moisture and water retention. Exhibit high antimicrobial properties.	[63]
PVA-CS nanofiber mats	Electrospun	Wound healing	Non-toxic to normal human fibroblast cells. Satisfactory antibacterial activity against *Staphylococcus aureus* and *Escherichia coli.* Showed greatest wound-healing activity during the first four days after wounds.	[64]

**Table 2:** Summary of the PVA-CS applications in other biomedical applications.

Composites	Methods	Applications	Advantages	Ref
PVA-oxidized CS-silver Nanoparticles/Ibuprofen film	Cross-linked	Periodontal pockets	The film has good antimicrobial properties against *Staphylococcus aureus*, *Klebsiella pneumoniae*, *Pseudomonas aeruginosa*, and *Porphyromonas gingivalis gingivalis*. Biocompatible as demonstrated by in vitro on HDFa cell lines.	[94]
PVA-CS-graphene oxide-astaxanthin nanofiber	Electrospun	Anti-inflammation and bone regeneration of periodontal therapy	Anti-inflammatory and osteogenic activity evaluated in vitro. Promote high expression of osteogenic genes OCN and Runx2 in BMSCs. Induce M2 polarization. Promote high expression of GPNMB in RAW264.7 cells. Glycoprotein nonmetastatic melanoma protein.	[95]
PVA-CS- Ofloxavin	Electrospun	Ocular drug delivery	Antimicrobial efficiency against *Staphylococcus aureus* and *Escherichia coli*. The use of Ofloxavin nanofibrous inserts on a rabbit eye demonstrated a sustained release pattern for up to 96 h. Demonstrates the potential of the nanofiber technology to sustain drug release in ocular drug delivery systems.	[96]
PVA-CS corneal shield	Electrospun	Ocular surface disorder	In vivo study demonstrated that the corneal shield applied to the rabbit eyes has good biocompatibility and drug delivery effect. No inflammatory reaction. No corneal edema.	[97]
PVA-CS-Silver/PVA-CS-Gold	Gamma-irradiated	Prostatic cancer	Significant effectiveness against prostatic cancer.	[98]
PVA-CS-Tea tree oil film	Emulsion	Biomedical application	Better resorption and biocompatibility. The incorporation of oil stimulates significant interaction with phagocytic cells.	[99]

## Data Availability

Not applicable.
